# 9-Ethyl-3-(2-methyl­benzo­yl)-9*H*-carbazole

**DOI:** 10.1107/S1600536809000737

**Published:** 2009-01-10

**Authors:** Ye-Chao Hang, Min-Dong Chen, Yong Wang

**Affiliations:** a College of Environmental Science and Engineering, Nanjing University of Information Science and Technology, Nanjing 210044, People’s Republic of China; b School of Pharmaceutical and Chemical Engineering, Taizhou University, Linhai 317000, People’s Republic of China

## Abstract

In the title compound, C_22_H_19_NO, the dihedral angle between the benzene ring and the carbazole ring system 77.1 (1)°.. The crystal structure is stabilized by inter­molecular aromatic π–π inter­actions between the benzene ring and the pyrrole ring of the carbazole system of neighbouring mol­ecules [centroid–centroid distance = 3.617 (4) Å]. In addition, the crystal structure exhibits a weak inter­molecular C—H⋯π inter­action.

## Related literature

For the synthesis, see Feng *et al.* (2007 ▶); For bond-length data, see: Allen *et al.* (1987 ▶). For background, see: Bai *et al.* (2007 ▶); Promarak *et al.* (2007 ▶); Liu *et al.* (2009 ▶).
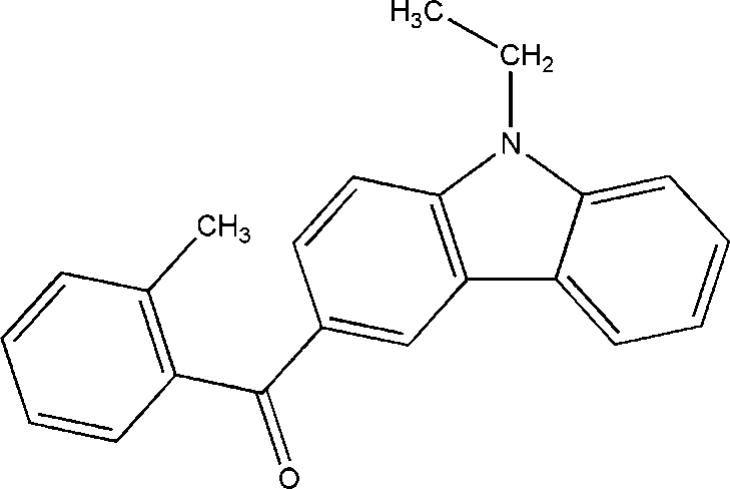


## Experimental

### 

#### Crystal data

C_22_H_19_NO
*M* *_r_* = 313.38
Monoclinic, 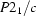

*a* = 11.676 (2) Å
*b* = 10.569 (2) Å
*c* = 13.756 (3) Å
β = 96.48 (3)°
*V* = 1686.7 (6) Å^3^
*Z* = 4
Mo *K*α radiation
μ = 0.08 mm^−1^
*T* = 298 (2) K
0.30 × 0.20 × 0.10 mm

#### Data collection

Enraf–Nonius CAD-4 diffractometer
Absorption correction: ψ scan (North *et al.*, 1968 ▶) *T*_min_ = 0.978, *T*_max_ = 0.993
3053 measured reflections
3053 independent reflections
2046 reflections with *I* > 2σ(*I*)
3 standard reflections every 200 reflections intensity decay: 1%

#### Refinement

*R*[*F* ^2^ > 2σ(*F* ^2^)] = 0.066
*wR*(*F* ^2^) = 0.173
*S* = 1.08
3053 reflections
217 parameters
H-atom parameters constrained
Δρ_max_ = 0.37 e Å^−3^
Δρ_min_ = −0.32 e Å^−3^

### 

Data collection: *CAD-4 Software* (Enraf–Nonius, 1985 ▶); cell refinement: *CAD-4 Software*; data reduction: *XCAD4* (Harms & Wocadlo, 1995 ▶); program(s) used to solve structure: *SHELXS97* (Sheldrick, 2008 ▶); program(s) used to refine structure: *SHELXL97* (Sheldrick, 2008 ▶); molecular graphics: *SHELXTL* (Sheldrick, 2008 ▶); software used to prepare material for publication: *SHELXTL*.

## Supplementary Material

Crystal structure: contains datablocks I, global. DOI: 10.1107/S1600536809000737/lx2084sup1.cif

Structure factors: contains datablocks I. DOI: 10.1107/S1600536809000737/lx2084Isup2.hkl

Additional supplementary materials:  crystallographic information; 3D view; checkCIF report

## Figures and Tables

**Table 1:** Hydrogen-bond geometry (Å, °)

*D*—H⋯*A*	*D*—H	H⋯*A*	*D*⋯*A*	*D*—H⋯*A*
C22—H22*B*⋯*Cg*3^i^	0.96	2.71	3.501 (4)	140

Symmetry codes: (i) 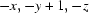. *Cg*3 is the centroids of the C16–C21 benzene ring.

**Fig. 1.**
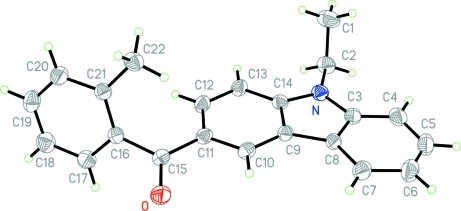
The molecular structure of the title compound, showing displacement ellipsoids drawn at the 30% probability level.

**Fig. 2.**
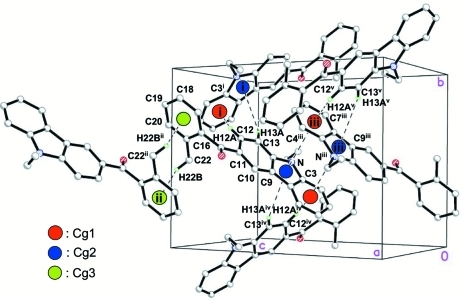
π—π and C—H···π interactions (dotted lines) in the title compound. Cg denotes ring centroid. [Symmetry code: (i) -x+1, y-1/2, -z+1/2; (ii) -x, -y+1, -z (iii) -x+1, -y+1, -z+1; (iv) -x+1, y+1/2, -z+1/2; (v) x, -y+1/2, z+1/2.]

## supplementary crystallographic information

### Comment

The title compound is one of important intermediates in synthesis of carbazole-containing compounds used as organic optoelectronic materials which have the characteristic of large π—π conjugation bones (Bai *et al.* 2007; Promarak *et al.* 2007; Liu *et al.* 2009). Here we report the crystal structure of the title compound, 9-ethyl-3-(2-methylbenzoyl)carbazole (Fig. 1).

In the title compound, the bond lengths and angles are within normal ranges (Allen *et al.*,  1987). Rings of A (C3–8), B (C3/C8/C9/C14/N), C (C9–14) and D (C16–21) are approximately plane, and dihedral angles of the four rings are: A/B = 1.1 (1)°, B/C = 2.6 (1)°, C/D = 76.0 (2)°. The crystals structure is stabilized by intermolecular aromatic π—π interactions between the benzene ring and the pyrrole ring of the carbazole system of neighbouring molecules. The Cg1···Cg2^iii^ distance is 3.617 (4) Å (Fig. 2; Cg1 and Cg2 are the centroids of the C3–C8 benzene ring and the C3/C8/C9/C14/N pyrrole ring, respectively, symmetry code as in Fig. 2). The molecular packing is further stabilized by intermolecular C—H···π interactions ; one between the hydrogen on the benzene ring and the benzene ring of neighbouring molecules (C12—H12A···Cg1^i^), a second the hydrogen on the benzene ring and the pyrrole ring of neighbouring molecules (C13—H13A···Cg2^i^), a third between the hydrogen of C–22 methyl group and the benzene ring of neighbouring molecules (C22—H22B···Cg3^ii^) (Fig. 2 and Table 1; Cg3 is the centroids of the C16–C21 benzene ring, symmetry code as in Fig. 2).

### Experimental

The title compound, (I) was prepared by a silimar method reported in literature (Feng *et al.*, 2007) with some modification. The crystals were obtained by dissolving I (0.15 g) in a mixture solvent of methanol (30 ml) and dichloromethane (20 ml) and evaporating the solvent slowly at room temperature for about 2 d.

### Refinement

H atoms were positioned geometrically, with O—H = 0.82 and C—H = 0.93 Å for aromatic H, and constrained to ride on their parent atoms, with *U*_iso_(H) = *xU*_eq_(C/O), where *x* = 1.2 for aromatic H and *x* = 1.5 for other H.

### Crystal data

**Table:**

C_22_H_19_NO	*F*(000) = 664
*M**_r_* = 313.38	*D*_x_ = 1.234 Mg m^−^^3^
Monoclinic, *P*2_1_/*c*	Mo *K*α radiation, λ = 0.71073 Å
Hall symbol: -P 2ybc	Cell parameters from 25 reflections
*a* = 11.676 (2) Å	θ = 9–13°
*b* = 10.569 (2) Å	µ = 0.08 mm^−^^1^
*c* = 13.756 (3) Å	*T* = 298 K
β = 96.48 (3)°	Plate, colorless
*V* = 1686.7 (6) Å^3^	0.30 × 0.20 × 0.10 mm
*Z* = 4	

### Data collection

**Table:**

Enraf–Nonius CAD-4 diffractometer	2046 reflections with *I* > 2σ(*I*)
Radiation source: fine-focus sealed tube	*R*_int_ = 0.0000
graphite	θ_max_ = 25.3°, θ_min_ = 1.8°
ω/2θ scans	*h* = −14→13
Absorption correction: ψ scan (North *et al.*, 1968)	*k* = 0→12
*T*_min_ = 0.978, *T*_max_ = 0.993	*l* = 0→16
3053 measured reflections	3 standard reflections every 200 reflections
3053 independent reflections	intensity decay: 1%

### Refinement

**Table:**

Refinement on *F*^2^	Primary atom site location: structure-invariant direct methods
Least-squares matrix: full	Secondary atom site location: difference Fourier map
*R*[*F*^2^ > 2σ(*F*^2^)] = 0.066	Hydrogen site location: difference Fourier map
*wR*(*F*^2^) = 0.173	H-atom parameters constrained
*S* = 1.08	*w* = 1/[σ^2^(*F*_o_^2^) + (0.0514*P*)^2^ + 1.9857*P*] where *P* = (*F*_o_^2^ + 2*F*_c_^2^)/3
3053 reflections	(Δ/σ)_max_ < 0.001
217 parameters	Δρ_max_ = 0.37 e Å^−^^3^
0 restraints	Δρ_min_ = −0.32 e Å^−^^3^

### Special details

**Table:**

Geometry. All e.s.d.'s (except the e.s.d. in the dihedral angle between two l.s. planes) are estimated using the full covariance matrix. The cell e.s.d.'s are taken into account individually in the estimation of e.s.d.'s in distances, angles and torsion angles; correlations between e.s.d.'s in cell parameters are only used when they are defined by crystal symmetry. An approximate (isotropic) treatment of cell e.s.d.'s is used for estimating e.s.d.'s involving l.s. planes.
Refinement. Refinement of *F*^2^ against ALL reflections. The weighted *R*-factor *wR* and goodness of fit *S* are based on *F*^2^, conventional *R*-factors *R* are based on *F*, with *F* set to zero for negative *F*^2^. The threshold expression of *F*^2^ > σ(*F*^2^) is used only for calculating *R*-factors(gt) *etc*. and is not relevant to the choice of reflections for refinement. *R*-factors based on *F*^2^ are statistically about twice as large as those based on *F*, and *R*- factors based on ALL data will be even larger.

### Fractional atomic coordinates and isotropic or equivalent isotropic displacement parameters (Å^2^)

**Table:**

	*x*	*y*	*z*	*U*_iso_*/*U*_eq_	
O	0.04033 (19)	0.4866 (2)	0.24020 (17)	0.0606 (7)	
N	0.5747 (2)	0.5162 (3)	0.36517 (18)	0.0451 (6)	
C1	0.7412 (3)	0.4981 (4)	0.2716 (3)	0.0768 (12)	
H1A	0.8113	0.4515	0.2686	0.115*	
H1B	0.7588	0.5864	0.2808	0.115*	
H1C	0.6917	0.4868	0.2116	0.115*	
C2	0.6818 (3)	0.4509 (4)	0.3554 (2)	0.0554 (9)	
H2A	0.6661	0.3613	0.3465	0.066*	
H2B	0.7330	0.4610	0.4155	0.066*	
C3	0.5588 (3)	0.6168 (3)	0.4282 (2)	0.0456 (8)	
C4	0.6411 (3)	0.6748 (4)	0.4954 (2)	0.0576 (9)	
H4A	0.7177	0.6492	0.5033	0.069*	
C5	0.6016 (4)	0.7725 (4)	0.5493 (3)	0.0660 (11)	
H5A	0.6532	0.8118	0.5963	0.079*	
C6	0.4896 (4)	0.8141 (3)	0.5365 (3)	0.0640 (10)	
H6A	0.4681	0.8827	0.5727	0.077*	
C7	0.4076 (3)	0.7551 (3)	0.4703 (2)	0.0549 (9)	
H7A	0.3311	0.7817	0.4630	0.066*	
C8	0.4436 (3)	0.6549 (3)	0.4150 (2)	0.0402 (7)	
C9	0.3850 (2)	0.5716 (3)	0.3427 (2)	0.0393 (7)	
C10	0.2712 (3)	0.5577 (3)	0.3037 (2)	0.0414 (7)	
H10A	0.2149	0.6117	0.3228	0.050*	
C11	0.2415 (2)	0.4626 (3)	0.2359 (2)	0.0414 (7)	
C12	0.3274 (3)	0.3846 (3)	0.2051 (2)	0.0458 (8)	
H12A	0.3070	0.3236	0.1575	0.055*	
C13	0.4413 (3)	0.3958 (3)	0.2433 (2)	0.0451 (7)	
H13A	0.4976	0.3423	0.2235	0.054*	
C14	0.4691 (2)	0.4903 (3)	0.3127 (2)	0.0395 (7)	
C15	0.1190 (3)	0.4411 (3)	0.1988 (2)	0.0433 (7)	
C16	0.0876 (2)	0.3582 (3)	0.1113 (2)	0.0380 (7)	
C17	0.0254 (3)	0.2480 (3)	0.1238 (2)	0.0511 (8)	
H17A	0.0064	0.2274	0.1857	0.061*	
C18	−0.0088 (3)	0.1689 (3)	0.0462 (3)	0.0604 (9)	
H18A	−0.0489	0.0948	0.0560	0.072*	
C19	0.0167 (3)	0.2000 (3)	−0.0451 (3)	0.0588 (9)	
H19A	−0.0060	0.1474	−0.0979	0.071*	
C20	0.0767 (3)	0.3106 (3)	−0.0587 (2)	0.0524 (8)	
H20A	0.0914	0.3322	−0.1216	0.063*	
C21	0.1159 (2)	0.3909 (3)	0.0190 (2)	0.0421 (7)	
C22	0.1797 (3)	0.5092 (3)	0.0004 (2)	0.0508 (8)	
H22A	0.2030	0.5510	0.0613	0.076*	
H22B	0.1305	0.5642	−0.0412	0.076*	
H22C	0.2466	0.4884	−0.0310	0.076*	

### Atomic displacement parameters (Å^2^)

**Table:**

	*U*^11^	*U*^22^	*U*^33^	*U*^12^	*U*^13^	*U*^23^
O	0.0475 (13)	0.0765 (18)	0.0590 (14)	0.0061 (12)	0.0109 (11)	−0.0164 (13)
N	0.0360 (13)	0.0548 (17)	0.0444 (14)	0.0025 (12)	0.0042 (11)	0.0022 (13)
C1	0.055 (2)	0.101 (3)	0.079 (3)	0.000 (2)	0.022 (2)	0.010 (2)
C2	0.0350 (17)	0.073 (2)	0.057 (2)	0.0078 (16)	−0.0005 (14)	0.0077 (18)
C3	0.0535 (19)	0.0459 (18)	0.0378 (16)	−0.0090 (15)	0.0062 (14)	0.0069 (14)
C4	0.061 (2)	0.066 (2)	0.0453 (18)	−0.0179 (19)	0.0015 (16)	0.0135 (18)
C5	0.082 (3)	0.065 (3)	0.048 (2)	−0.029 (2)	−0.0035 (19)	−0.0012 (19)
C6	0.086 (3)	0.047 (2)	0.058 (2)	−0.012 (2)	0.004 (2)	−0.0085 (18)
C7	0.069 (2)	0.0419 (19)	0.055 (2)	−0.0072 (17)	0.0134 (17)	−0.0039 (16)
C8	0.0474 (17)	0.0347 (16)	0.0390 (15)	−0.0078 (13)	0.0067 (13)	0.0000 (13)
C9	0.0436 (16)	0.0357 (16)	0.0392 (15)	−0.0034 (13)	0.0080 (13)	−0.0042 (13)
C10	0.0467 (17)	0.0364 (16)	0.0417 (16)	0.0084 (14)	0.0076 (13)	−0.0028 (13)
C11	0.0424 (17)	0.0423 (18)	0.0400 (16)	0.0050 (14)	0.0070 (13)	−0.0048 (14)
C12	0.0475 (18)	0.0461 (18)	0.0438 (17)	0.0018 (15)	0.0047 (14)	−0.0114 (15)
C13	0.0447 (17)	0.0450 (18)	0.0459 (17)	0.0078 (14)	0.0061 (14)	−0.0102 (15)
C14	0.0365 (15)	0.0432 (17)	0.0393 (15)	0.0024 (13)	0.0060 (12)	0.0016 (13)
C15	0.0465 (17)	0.0463 (18)	0.0375 (15)	0.0032 (15)	0.0071 (14)	−0.0018 (14)
C16	0.0338 (14)	0.0382 (16)	0.0411 (15)	0.0042 (13)	0.0006 (12)	0.0021 (13)
C17	0.058 (2)	0.0434 (19)	0.0521 (18)	−0.0050 (16)	0.0080 (16)	0.0064 (16)
C18	0.065 (2)	0.046 (2)	0.070 (2)	−0.0097 (17)	0.0068 (19)	−0.0054 (18)
C19	0.063 (2)	0.053 (2)	0.058 (2)	−0.0001 (18)	−0.0026 (18)	−0.0151 (18)
C20	0.0509 (19)	0.061 (2)	0.0444 (17)	0.0020 (17)	0.0023 (15)	−0.0029 (16)
C21	0.0371 (16)	0.0461 (18)	0.0432 (16)	0.0034 (13)	0.0043 (13)	0.0011 (14)
C22	0.0528 (19)	0.0474 (19)	0.0525 (19)	−0.0042 (15)	0.0066 (15)	0.0100 (16)

### Geometric parameters (Å, °)

**Table:**

O—C15	1.231 (3)	C10—C11	1.388 (4)
N—C14	1.384 (4)	C10—H10A	0.9300
N—C3	1.397 (4)	C11—C12	1.400 (4)
N—C2	1.448 (4)	C11—C15	1.481 (4)
C1—C2	1.497 (5)	C12—C13	1.380 (4)
C1—H1A	0.9600	C12—H12A	0.9300
C1—H1B	0.9600	C13—C14	1.394 (4)
C1—H1C	0.9600	C13—H13A	0.9300
C2—H2A	0.9700	C15—C16	1.501 (4)
C2—H2B	0.9700	C16—C17	1.392 (4)
C3—C4	1.397 (4)	C16—C21	1.392 (4)
C3—C8	1.397 (4)	C17—C18	1.379 (5)
C4—C5	1.380 (5)	C17—H17A	0.9300
C4—H4A	0.9300	C18—C19	1.363 (5)
C5—C6	1.373 (5)	C18—H18A	0.9300
C5—H5A	0.9300	C19—C20	1.385 (5)
C6—C7	1.393 (5)	C19—H19A	0.9300
C6—H6A	0.9300	C20—C21	1.401 (4)
C7—C8	1.396 (4)	C20—H20A	0.9300
C7—H7A	0.9300	C21—C22	1.492 (4)
C8—C9	1.441 (4)	C22—H22A	0.9600
C9—C10	1.385 (4)	C22—H22B	0.9600
C9—C14	1.401 (4)	C22—H22C	0.9600
			
C14—N—C3	107.6 (2)	C10—C11—C12	119.8 (3)
C14—N—C2	125.8 (3)	C10—C11—C15	120.0 (3)
C3—N—C2	126.6 (3)	C12—C11—C15	120.2 (3)
C2—C1—H1A	109.5	C13—C12—C11	121.8 (3)
C2—C1—H1B	109.5	C13—C12—H12A	119.1
H1A—C1—H1B	109.5	C11—C12—H12A	119.1
C2—C1—H1C	109.5	C12—C13—C14	117.5 (3)
H1A—C1—H1C	109.5	C12—C13—H13A	121.2
H1B—C1—H1C	109.5	C14—C13—H13A	121.2
N—C2—C1	113.1 (3)	N—C14—C13	128.6 (3)
N—C2—H2A	109.0	N—C14—C9	109.7 (3)
C1—C2—H2A	109.0	C13—C14—C9	121.7 (3)
N—C2—H2B	109.0	O—C15—C11	121.5 (3)
C1—C2—H2B	109.0	O—C15—C16	118.1 (3)
H2A—C2—H2B	107.8	C11—C15—C16	120.3 (3)
N—C3—C4	128.0 (3)	C17—C16—C21	120.0 (3)
N—C3—C8	109.4 (3)	C17—C16—C15	118.3 (3)
C4—C3—C8	122.6 (3)	C21—C16—C15	121.7 (3)
C5—C4—C3	116.0 (3)	C18—C17—C16	121.4 (3)
C5—C4—H4A	122.0	C18—C17—H17A	119.3
C3—C4—H4A	122.0	C16—C17—H17A	119.3
C6—C5—C4	122.8 (3)	C19—C18—C17	119.5 (3)
C6—C5—H5A	118.6	C19—C18—H18A	120.2
C4—C5—H5A	118.6	C17—C18—H18A	120.2
C5—C6—C7	121.0 (4)	C18—C19—C20	119.6 (3)
C5—C6—H6A	119.5	C18—C19—H19A	120.2
C7—C6—H6A	119.5	C20—C19—H19A	120.2
C6—C7—C8	118.0 (3)	C19—C20—C21	122.3 (3)
C6—C7—H7A	121.0	C19—C20—H20A	118.8
C8—C7—H7A	121.0	C21—C20—H20A	118.8
C7—C8—C3	119.6 (3)	C16—C21—C20	117.0 (3)
C7—C8—C9	133.7 (3)	C16—C21—C22	122.7 (3)
C3—C8—C9	106.7 (3)	C20—C21—C22	120.2 (3)
C10—C9—C14	119.6 (3)	C21—C22—H22A	109.5
C10—C9—C8	133.8 (3)	C21—C22—H22B	109.5
C14—C9—C8	106.6 (3)	H22A—C22—H22B	109.5
C9—C10—C11	119.6 (3)	C21—C22—H22C	109.5
C9—C10—H10A	120.2	H22A—C22—H22C	109.5
C11—C10—H10A	120.2	H22B—C22—H22C	109.5
			
C14—N—C2—C1	82.1 (4)	C2—N—C14—C13	2.5 (5)
C3—N—C2—C1	−97.4 (4)	C3—N—C14—C9	−1.2 (3)
C14—N—C3—C4	179.7 (3)	C2—N—C14—C9	179.2 (3)
C2—N—C3—C4	−0.7 (5)	C12—C13—C14—N	176.2 (3)
C14—N—C3—C8	−0.4 (3)	C12—C13—C14—C9	−0.2 (5)
C2—N—C3—C8	179.2 (3)	C10—C9—C14—N	−176.4 (3)
N—C3—C4—C5	−179.6 (3)	C8—C9—C14—N	2.2 (3)
C8—C3—C4—C5	0.5 (5)	C10—C9—C14—C13	0.6 (4)
C3—C4—C5—C6	−2.0 (5)	C8—C9—C14—C13	179.2 (3)
C4—C5—C6—C7	2.8 (6)	C10—C11—C15—O	−15.2 (5)
C5—C6—C7—C8	−2.0 (5)	C12—C11—C15—O	162.8 (3)
C6—C7—C8—C3	0.5 (5)	C10—C11—C15—C16	167.3 (3)
C6—C7—C8—C9	178.6 (3)	C12—C11—C15—C16	−14.7 (4)
N—C3—C8—C7	−179.7 (3)	O—C15—C16—C17	−60.7 (4)
C4—C3—C8—C7	0.2 (5)	C11—C15—C16—C17	116.9 (3)
N—C3—C8—C9	1.8 (3)	O—C15—C16—C21	117.3 (3)
C4—C3—C8—C9	−178.3 (3)	C11—C15—C16—C21	−65.2 (4)
C7—C8—C9—C10	−2.4 (6)	C21—C16—C17—C18	0.5 (5)
C3—C8—C9—C10	175.9 (3)	C15—C16—C17—C18	178.5 (3)
C7—C8—C9—C14	179.3 (3)	C16—C17—C18—C19	−1.3 (5)
C3—C8—C9—C14	−2.4 (3)	C17—C18—C19—C20	0.1 (5)
C14—C9—C10—C11	0.6 (4)	C18—C19—C20—C21	2.1 (5)
C8—C9—C10—C11	−177.5 (3)	C17—C16—C21—C20	1.5 (4)
C9—C10—C11—C12	−2.2 (4)	C15—C16—C21—C20	−176.4 (3)
C9—C10—C11—C15	175.7 (3)	C17—C16—C21—C22	178.4 (3)
C10—C11—C12—C13	2.8 (5)	C15—C16—C21—C22	0.5 (4)
C15—C11—C12—C13	−175.2 (3)	C19—C20—C21—C16	−2.8 (5)
C11—C12—C13—C14	−1.5 (5)	C19—C20—C21—C22	−179.8 (3)
C3—N—C14—C13	−177.9 (3)		

### Hydrogen-bond geometry (Å, °)

**Table:**

*D*—H···*A*	*D*—H	H···*A*	*D*···*A*	*D*—H···*A*
C12—H12A···Cg1^i^	0.93	3.13	3.730 (4)	124
C13—H13A···Cg2^i^	0.93	3.18	3.928 (4)	138
C22—H22B···Cg3^ii^	0.96	2.71	3.501 (4)	140

Symmetry codes: (i) −*x*+1, *y*−1/2, −*z*+1/2; (ii) −*x*, −*y*+1, −*z*.
